# IRMM-1000a and IRMM-1000b: uranium reference materials certified for the production date based on the ^230^Th/^234^U radiochronometer. Part II: certification

**DOI:** 10.1007/s10967-015-4368-y

**Published:** 2015-09-04

**Authors:** C. Venchiarutti, Z. Varga, S. Richter, A. Nicholl, J. Krajko, R. Jakopič, K. Mayer, Y. Aregbe

**Affiliations:** European Commission, Joint Research Centre (JRC), Institute for Reference Materials and Measurements (IRMM), Retieseweg 111, 2440 Geel, Belgium; European Commission, Joint Research Centre (JRC), Institute for Transuranium Elements (ITU), Postfach 2340, 76125 Karlsruhe, Germany

**Keywords:** CRMs, Uranium age-dating, ^230^Th/^234^U radiochronometer, Certified reference material, Nuclear safeguards and forensics

## Abstract

The IRMM-1000a and IRMM-1000b uranium reference materials, of 20 and 50 mg uranium, respectively, were produced by the European Commission Joint Research Centre’s Institute for Reference Materials and Measurements (EC-JRC-IRMM) in collaboration with the Institute for Transuranium Elements (EC-JRC-ITU). They are novel uranium reference materials certified for the production date based on the ^230^Th/^234^U radiochronometer, i.e. the date of the last chemical separation of these two radionuclides. The certified reference value and its uncertainty, homogeneity and stability of the material were established in accordance with the ISO Guide 34:2009 and the ‘Guide to the Expression of Uncertainty in Measurement’.

## Introduction

The Institute for Reference Materials and Measurements of the Joint Research Centre of the European Commission (EC-JRC-IRMM) is an accredited provider of certified reference materials (CRMs) produced according to ISO Guide 34 [1]. The EC-JRC-IRMM in collaboration with the Institute for Transuranium Elements of the Joint Research Centre of the European Commission (EC-JRC-ITU) produced novel uranium reference materials IRMM-1000a and IRMM-1000b certified for the production date based on the ^230^Th/^234^U radiochronometer. The production date corresponds to the date of the last chemical separation and removal, to the maximum extent achievable, of ^230^Th from ^234^U in the reference material. From that date, the ^230^Th present in the material is solely due to decay of ^234^U. Therefore by measuring the *n*(^230^Th)/*n*(^234^U) amount ratio or *A*(^230^Th)/*A*(^234^U) activity ratio, the age of the material can be determined and compared to the certified production date of the IRMM-1000a and IRMM-1000b. Such CRMs together with advanced analytical methods [2], are required to characterise intercepted nuclear materials and to identify without ambiguity their origins.

This paper is complimentary to the paper describing the preparation of the candidate uranium reference materials by Varga et al. [3]. It describes the specific approach compliant with ISO Guide 34:2009 that needed to be developed for the certification of IRMM-1000a and IRMM-1000b. The CRMs were prepared at EC-JRC-ITU from a low-enriched uranium solution after complete separation of thorium impurities, i.e. removing the initial daughter nuclide concentration at a well-known time. The completeness of the separation was monitored by γ-ray spectrometry and by inductively coupled mass spectrometry (ICP–MS). The reference value (production date) of the IRMM-1000a and IRMM-1000b corresponds to the date and carefully recorded time of the last separation of ^230^Th from ^234^U, which took place on 9 July, 2012. Aliquots of the purified uranium mother solution were dispensed into single units of two different sizes, 20 and 50 mg, as dried uranyl nitrate corresponding to IRMM-1000a and IRMM-1000b respectively [3].

Part of the certification process is the assignment of a reference value and its associated uncertainty [1–4]. The certification process consists of the characterisation, homogeneity and stability studies:

- The characterisation study, which consists of the assessment of the selectivity, absence of interferences and method correctness and results in the determination of the reference value (in this case the recorded production date) and the uncertainty from the characterisation study, noted *u*_char_.
- The homogeneity study to quantify the variations between and within units resulting in the determination of the uncertainty from homogeneity assessment *u*_bb_ using the standard deviation between units *s*_bb_.
- The stability study to check the stability of the material with time and determine the uncertainty associated with the reproducibility of the measurements *u*_lts_ (long-term stability).

The full uncertainty budget is established in accordance with the ‘Guide to the Expression of Uncertainty in Measurement’ [5] and the assigned uncertainty of the reference value *u*_CRM_ (expanded uncertainty) comprising the contributions from the three studies is expressed as follows:

1 $$u_{\text{CRM}} = k\;{ \times }\;\sqrt {u_{\text{char}}^{2} + u_{\text{bb}}^{2} + u_{\text{lts}}^{2} }$$

where *u*_char_, *u*_bb_ and *u*_lts_ are the combined standard uncertainties of the characterisation, homogeneity and long-term stability assessments with the coverage factor *k* according to the confidence level of the assigned uncertainty (in general, CRMs uncertainties are reported with *k* = 2, i.e. a 95 % CI).

In this paper, the results of the characterisation, homogeneity and stability studies on the IRMM-1000a and IRMM-1000b are presented and discussed.

## Characterisation and confirmation

The characterisation of a material according to ISO Guide 35 [6] is the process of determining the property value of the reference material. In the case of the IRMM-1000a and IRMM-1000b, the reference value was assigned on the basis of the reading of the clock at the time of the last chemical separation, i.e. carefully recording the date and time when ^230^Th was completely removed from its “parent”—^234^U—from the original uranium nitrate material. The reference value is identical for both IRMM-1000a and IRMM-1000b since the same purified mother solution was used for aliquoting of the two different sizes [3].

The reference value for this CRM is 9 July, 2012 at 11:08 a.m. [3], expressed as 09/07/2012 ± *u*_CRM_ in day. The final uncertainty *u*_CRM_ of the IRMM-1000a and IRMM-1000b is traceable to the international system of units (SI) via the universal coordinated time (UTC).

The combined standard uncertainty inherent to the production of the reference material, i.e. to the characterisation study, includes firstly the uncertainty on the exact time of the last chemical separation of Th from the U material. This time was estimated as the median of the starting and finishing times of the thorium elution in order to account for the whole thorium elution time and corresponds to 1.5 h (90 min or 0.063 day, *k* = 1). Secondly, it takes into account the uncertainty coming from the residual ^230^Th in the reference material during its production, which was found to be less than 80 min or 0.056 day (*k* = 1) [3]. The combined standard uncertainty for the characterisation is therefore 0.084 day (*k* = 1).

Confirmation measurements were carried out at EC-JRC-ITU in compliance with ISO Guide 34 [1] to assess if the measured age corresponded to the known production date. Confirmation measurements were performed by isotope dilution mass spectrometry (IDMS) analysis on randomly selected units in order to determine the *n*(^230^Th)/*n*(^234^U) amount ratio in the samples. The simplified IDMS equation [7] used to calculate either the ^230^Th or the ^234^U amount content (*C*_X_) in the sample is expressed as follows:

2 $$C_{\text{X}} = \frac{{\left( {R_{\text{Y}} - R_{\text{B}} } \right)}}{{\left( {R_{\text{B}} - R_{\text{X}} } \right)}}\; \times \;\frac{{m_{\text{Y}} }}{{m_{\text{X}} }}\; \times \;R_{\text{X}} \; \times \;C_{\text{Y}} ,$$

where *R*_X_, *R*_Y_, *R*_B_ represent the *n*(^230^Th)/*n*(^232^Th) or *n*(^234^U)/*n*(^233^U) amount ratio in the unknown sample, spike and blend, respectively, *m*_X_ and *m*_Y_ are the masses (in g) of unknown sample and spike, respectively and *C*_Y_ is the amount content of the spike (in mol g^−1^) (either ^232^Th or ^233^U).

The suprapur HNO_3_ was sub-boiled and all the chemical labware was cleaned prior to use [3].

Six 20 mg units were randomly selected from the whole batch and dissolved in 2 mL concentrated nitric acid. The total U concentration in these samples was 10 mg mL^−1^ and corresponded to an amount of about 1 pg of ^230^Th in each measured aliquot, almost 8 months after the production of the reference material.

Aliquots were prepared gravimetrically from the 2 mL dissolved samples and measured by thermal ionisation mass spectrometry (TIMS) on the MAT262 from Thermo Fischer Scientific, Bremen, Germany and ICP–MS on the Element 2 from Thermo Fischer Scientific, Bremen, Germany. An in-house ^233^U spike (3.5 μg g^−1^) and two natural ^232^Th spikes (one from Inorganic Ventures with a total Th concentration of [1001 ± 5] μg mL^−1^, *k* = 2, and one from SPEX CertiPrep with a total Th concentration of [1000 ± 5] μg g^−1^, noted Th#2 and Th#3 respectively) were used to determine the uranium and the thorium amount contents [3]. Aliquots for Th separations from the randomly selected units (referred as A, B, C, D, E and F) were prepared gravimetrically using ~0.1 g of solution and ~0.1 g of natural ^232^Th spike. In addition, a blank and an unspiked sample were prepared for each of the six aliquots. The aliquots were chemically purified by a single separation using TEVA® Resin following a similar measurement procedure as that described in [3]. The purified solutions were diluted in nitric acid and homogenised prior to the measurements using the Element 2 ICP–MS [3, 8].

Procedural blanks, Th standards and unspiked samples were measured before each measurement series.

From the measured *n*(^230^Th)/*n*(^234^U) amount ratio in the samples, the ages were calculated using the following simplified equation assuming complete Th separation [9]:

3 $$t = \frac{1}{{\lambda_{{{}^{234}{\text{U}}}} - \lambda_{{{}^{230}{\text{Th}}}} }}\; \times \;\ln \left( {1 - \frac{{n({}^{230}{\text{Th}})}}{{n({}^{234}{\text{U}})}}\; \times \;\frac{{\lambda_{{{}^{230}{\text{Th}}}} - \lambda_{{{}^{234}{\text{U}}}} }}{{\lambda_{{{}^{234}{\text{U}}}} }}} \right)$$

where *t* is the age of the uranium sample (in years), $\lambda_{{{}^{234}{\text{U}}}}$ and $\lambda_{{{}^{230}{\text{Th}}}}$ are the decay constants of ^234^U and ^230^Th respectively, based on their respective half-lives, *T*_1/2_ = (245.5 ± 1.2)·10^3^*a* and *T*_1/2_ = (75.38 ± 0.6)·10^3^*a*, *k* = 2 [10]. The efficiency and completeness of the chemical separation was verified and is discussed in details in [3].

The detailed uncertainty contributions for the measurements of the *n*(^230^Th)/*n*(^232^Th) and *n*(^234^U)/*n*(^233^U) amount ratios and calculated ages are summarised in Table 1.

**Table 1:** Summary of the different uncertainty contributions (in %) on the thorium, uranium amount contents and the final calculated age for the three studies: confirmation, homogeneity and long-term stability

	Confirmation	Homogeneity	Long-term stability
Th measurements	*R*_B_ ≥ 75 % *C*_Y_ (^232^Th) ≤ 10 % Mass bias factor ≤ 10 % *m*_Y_ ≤ 5 %	*R*_B_ ≥ 70 % *C*_Y_ (^232^Th) and mass bias factor ≤ 10 % *m*_Y_ ≤ 10 % *R*_Y_ < 5 % *m*_X_ < 5 %	*R*_B_ ≤ 80 % Mass bias factor < 30 % *C*_Y_ (^232^Th) < 20 % *m*_Y_ ≤ 10 %
U measurements	*R*_B_ ≥ 80 % Mass bias factor ≤ 20 %	Mass bias factor ≤ 80 % *R*_B_ ≤ 10 %	*R*_B_ ≥ 60 % Mass bias factor ≤ 40 %
Age calculation	*C*_X_ (^230^Th) ≥ 50 % *C*_X_ (^234^U) ≤ 50 %	*C*_X_ (^230^Th) ≤ 50 % *C*_X_ (^234^U) ≥ 50 % Half-life of ^234^U ~ 5 %	*C*_X_ (^230^Th) > 60 % *C*_X_ (^234^U) ≤ 20 % Half-life of ^234^U ≤ 20 %

**Fig. 1:**
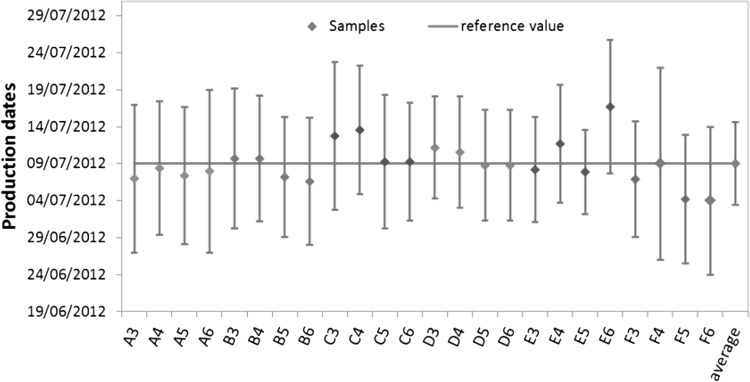
Production dates as calculated from the measured ages for the 24 samples (*diamond* symbols are colour coded according to six sample sets, A through F) for the confirmation study, the reference value (*horizontal line*) and their associated expanded uncertainties (*k* = 2) and the average and its uncertainty (as 2SD, standard deviation). The thorium IDMS samples are numbered as follows: 3–4 samples spiked with Th#2 and 5–6 spiked with Th#3

The measured production dates (in dd/mm/yyyy) with their expanded uncertainties (*k* = 2) are presented in Fig. 1 and are compared with the known production date of the reference material, i.e. 9 July, 2012.

Figure 1 shows a good agreement of the calculated production dates both with the average value and the known production date (reference value). It confirms the completeness of the separation of the thorium from the uranium during the production of the uranium reference material. Moreover; these results also show that there is no difference or bias observed using the two Th spikes for the IDMS analyses.

## Homogeneity

A key requirement for any reference material is the equivalence between the various units [6, 11]. In this respect, it is relevant whether the variation between units (heterogeneity) is significant compared to the uncertainty of the certified value. In contrast to that, it is not relevant if this variation between units is significant compared to the analytical variation. Consequently, ISO Guide 34 requires reference material producers to quantify the between unit variation/homogeneity.

The between-unit homogeneity was evaluated to ensure that the certified value of the uranium reference material is valid for the whole batch of 161 units within the stated uncertainty. Five units of 20 mg and five units of 50 mg uranium (corresponding to the cubic root of the produced unit number in each size) were selected using a random stratified sampling scheme covering the whole batch.

The same chemical preparation of the samples was used as for the characterisation and confirmation in Sect. “Characterisation and confirmation”. The ten selected units were dissolved in 2 or 5 mL concentrated nitric acid to obtain a U concentration of ~10 mg mL^−1^ and an amount of ~10 pg of ^230^Th per measured aliquot. Aliquots from the 2 or 5 mL were then prepared and measured by TIMS and ICP–MS.

For the uranium isotope abundance measurements, one aliquot per sample (so 10 aliquots in total) was prepared and measured by TIMS. For the determination of the ^234^U amount content using the *n*(^234^U)/*n*(^233^U) amount ratio by ID–ICP–MS (Element 2), one aliquot was prepared with an addition of the ^233^U spike (3.5 μg g^−1^) together with an unspiked aliquot. Procedural blanks were also measured by ICP–MS together with the uranium samples.

For the determination of the ^230^Th amount content, three aliquots per sample were prepared and spiked with the same natural ^232^Th tracer (i.e. Th#3) resulting in ten measurement series (hereafter referred with letters from A to J). An unspiked sample, procedural blanks and the Th#3 spike were also measured by ICP–MS.

The separation of the ten series was carried out on a single TEVA® Resin column on two consecutive days, starting with series A on the 16 October, 2013 at 14:40 p.m. (median time for complete Th elution), series B and series C on 17 October, 2013 at 11:00 a.m. and 14:50 p.m. respectively.

Although the thorium and uranium samples were not measured on the same day, day-to-day effects due to ingrowth of thorium are negligible due to the long half-lives of ^234^U and ^230^Th [10]. The thorium measurements were carried out under repeatability conditions and in a randomised manner to be able to separate a potential analytical drift from a trend in the filling sequence.

**Fig. 2:**
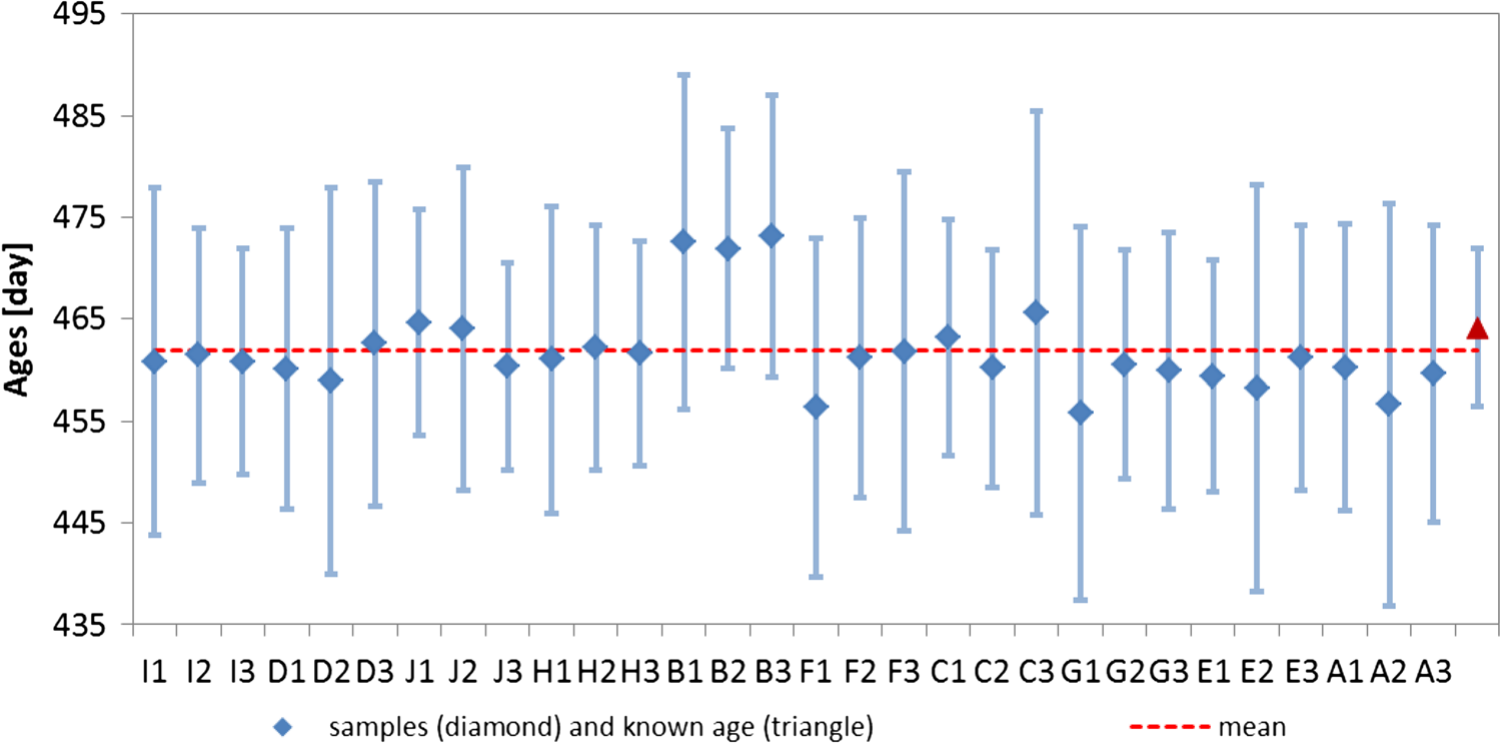
Calculated age obtained for the homogeneity study per unit in the analytical sequence order, the mean (*dashed line*) and the known age based on the reference value (*triangle*) with their expanded uncertainties (*k* = 2). Note that the uncertainty for the reference value corresponds to the uncertainties on the characterisation as described in Sect. “Characterisation and confirmation” and on the homogeneity as described in Sect. “Homogeneity”

The ages for the 10 selected units reported in Fig. 2 were normalised to the date of the separation of the first series (here 16 October, 2013 at 14:40 p.m. for series A), by subtracting the time difference between the separation date of the respective series and series A in order to compare all the ages. The known elapsed time since the production date of the material and the reference date of the analysis is hereafter referred to as the “known age”, and is 464.2 days. The Fig. 2 shows a good agreement within the uncertainties between the calculated (model) ages, the mean and the known age.

Regression analyses were performed to evaluate potential trends in the analytical sequence as well as trends in the filling sequence. No trends were observed either in the filling sequence or in the analytical sequence on a 95 % confidence interval (Fig. 2).

Quantification of between-unit inhomogeneity was accomplished by analysis of variance (ANOVA) using SoftCRM [12], which separates the between-unit variation (*s*_bb_) from the within-unit variation (*s*_wb_). The latter is equivalent to the method repeatability if the individual samples are representative for the whole unit [11]. Therefore, the data were checked for any significant difference in between-day means using one way- ANOVA on the samples in the analytical sequence order.

The dataset was tested for consistency using Grubbs outlier tests on a confidence level of 99 % on the individual results (replicate measurement) and the unit means. Two outlying individual results (Fig. 2), corresponding to two aliquots of the same unit (aliquots B1 and B3), were detected for the ages. The ages in these two aliquots were higher than in the other samples due to the higher ^232^Th measured in the procedural blank, thereby leading to an overcorrection of the ^232^Th signal and a higher ^230^Th/^232^Th isotope ratio. However all the data were retained for the statistical analysis, since no technical reason justified discarding these two individual results.

As summarised in Table 2, an inhomogeneity (*u*_bb,rel_^*^) of maximum 0.14 % was found whereas the between-unit variation (*s*_bb,rel_) was found to be of 0.83 %, and therefore above this limit to detect inhomogeneity. Therefore the between-unit standard deviation *s*_bb_ (0.83 %) can be used as estimate of the final uncertainty coming from the homogeneity study (*u*_bb_). The combined standard uncertainty to account for potential inhomogeneity is therefore *u*_*bb*_ = 3.88 day (*k* = *1*).

**Table 2:** Relative standard uncertainties on the age results from the homogeneity study carried out on 16 October 2013

	*s*_wb,rel_ (%)	*s*_bb,rel_ (%)	*u*_bb,rel_^*^ (%)	*u*_bb,rel_ (%)
Based on *n*(^230^Th)/*n*(^234^U)	0.43	0.83	0.14	0.83

## Stability

The IRMM-1000a and IRMM-1000b are certified for the production date based on the ^234^U/^230^Th radiochronometer. Therefore, the certified value is not subject to any instability by itself, and stability testing was only used to establish the reproducibility of the measurement procedure over time.

The long-term stability study [6, 11] was carried out more than two years after the production of the IRMM-1000a and IRMM-1000b units, following the same analytical procedures as the homogeneity and characterisation studies. The chemical separation took place on 3 November, 2014 at 10:53 a.m.

**Fig. 3:**
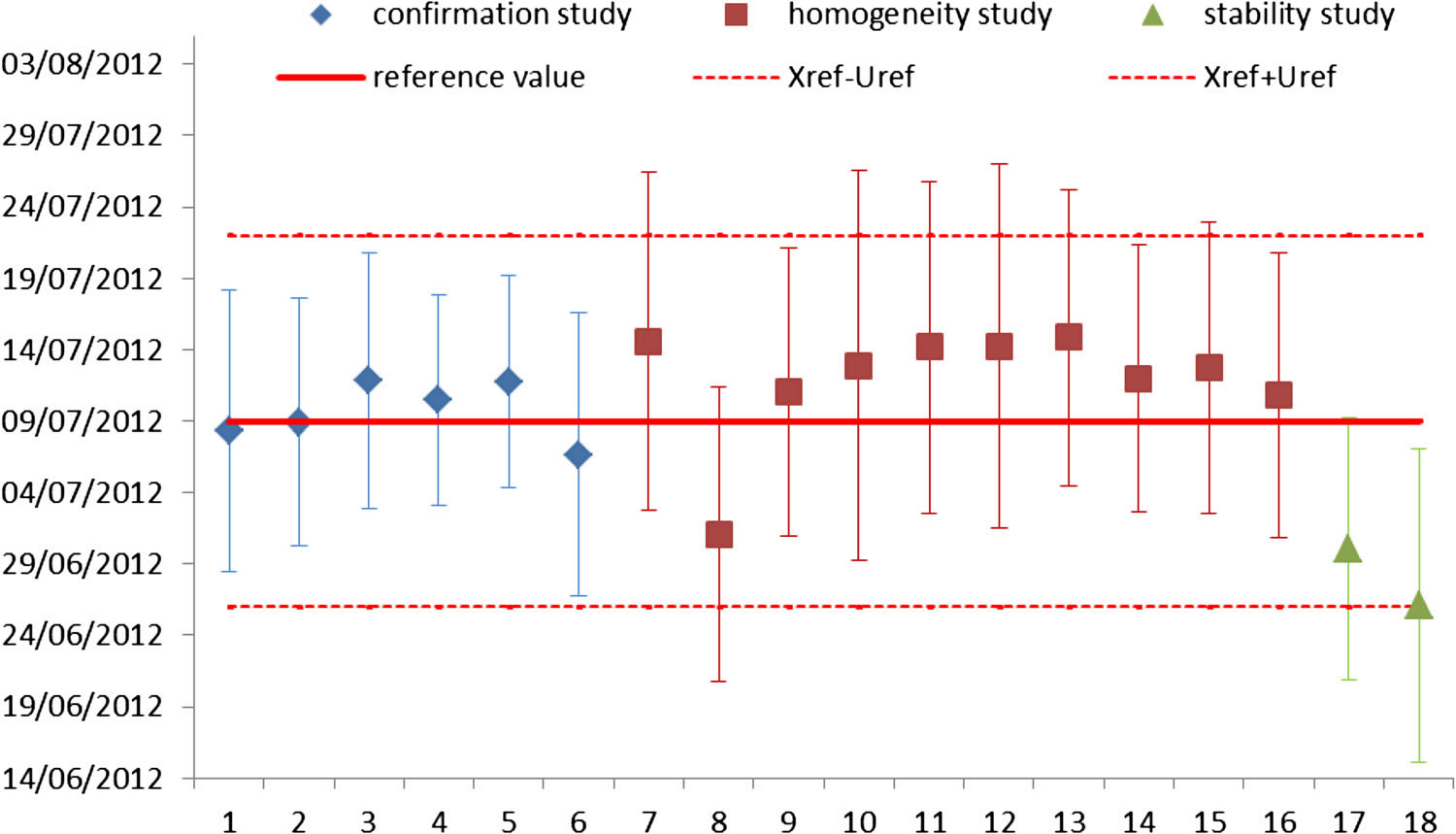
Calculated production dates and expanded uncertainties (*k* = 2) obtained from the three studies: characterisation (*diamonds*, on six units), homogeneity (*squares*, on ten units), stability study (*triangles*, on two units) and the certified reference value (*line*) with its expanded uncertainty (*dashed lines*)

Two 20 mg uranium samples (referred hereafter as A and B) were selected and analysed by ICP–MS using three aliquots for the thorium and two independent aliquots for the uranium analysis; these latter being also analysed by IDMS–TIMS. All the thorium aliquots were separated and measured by ICP–MS within one day and all the uranium aliquots the following day. Therefore, no normalisation to a common reference date was needed and the ages could directly be compared with the known age based on the ICP–MS results. The production dates for the two samples of the long-term stability study are presented along with the production dates from the two other studies in Fig. 3. They showed a good agreement within uncertainties with the certified reference value.

Because the number of samples for stability measurements was less than three, a conventional single and double Grubbs test at 99 % confidence level could not be performed to check for outliers. However, the results from the long term stability measurements were evaluated using Student test and *F* test (Table 3), which demonstrated that there was no trend and no outliers for a 99 % level of confidence and therefore, all data were retained for statistical analysis.

**Table 3:** Results from the Student test and *F* test on the six samples measured for the long-term stability study (two randomly selected units with three replicates per unit)

*t* value	4.47
*t* crit	4.60
*F*	5.67
*F* crit	7.71
df	4
*P* value	0.08

**Fig. 4:**
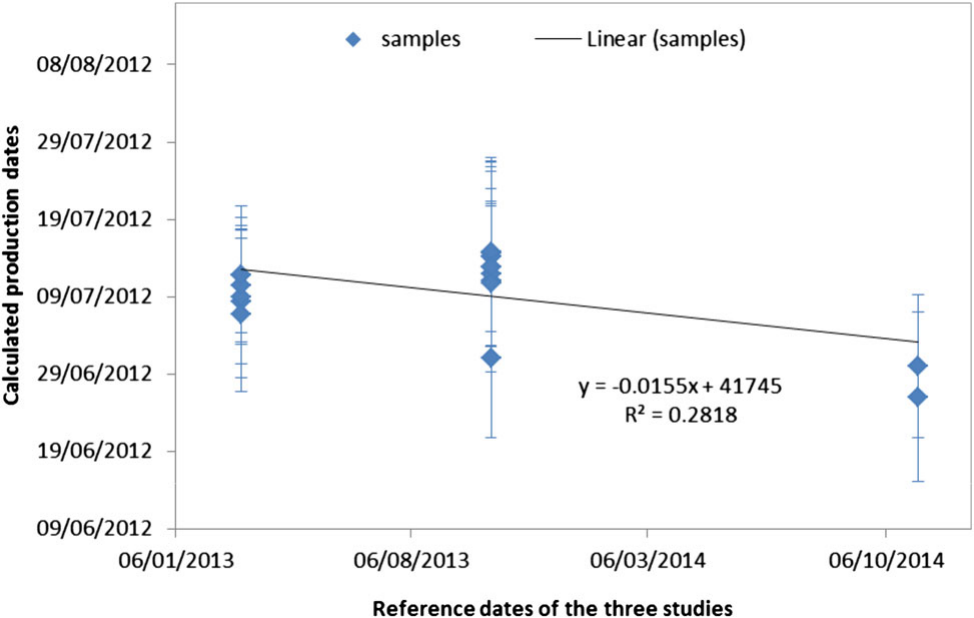
Results for the production dates (*N* = 18) with their expanded uncertainties (*k* = 2) at the respective dates of the confirmation, homogeneity and long-term stability studies

Furthermore, the data were plotted against the elapsed time between the confirmation and the long-term stability studies (i.e. taking into account all the results from the different certification studies) and linear regression lines of production dates versus time were calculated (as shown in Fig. 4). The slope of the regression line (*a* = −0.016 ± 0.007) was tested for statistical significance (loss/increase due to storage conditions) and was found to be significantly different from zero for a 95 % level of confidence, but not significantly different for a 99 % level of confidence. It was therefore concluded that there is no detectable degradation of the material. Since no technically unexplained outliers were observed and none of the trends were statistically significant for a 99 % level of confidence at room temperature, the material can be stored at room temperature.

The *u*_lts_ was calculated, in compliance with ISO Guide 35:2006 [6] based on the uncertainty of the slope of the regression line for the production dates from the three studies over time (Fig. 4) and a particular ‘shelf-life’, i.e. the storage time in days was derived to be *u*_lts_ = 5.20 day (*k* = 1) for a storage of 2 years (exactly 732 days) [6, 13]. The uncertainty from the long-term stability has therefore to be taken into account in the final combined uncertainty of the CRM (in Eq. 1).

As no significant degradation was found during this study, the CRM can be shipped to customers and stored for at least 2 years at ambient conditions without special precautions. However, this CRM will be subjected to EC-JRC-IRMM’s regular stability monitoring programme to monitor its stability beyond the 2 years.

## Final combined uncertainty

The assigned uncertainty consists of uncertainties related to characterisation, *u*_char_, potential between-unit inhomogeneity, *u*_bb_ and the uncertainty related to degradation during long-term storage *u*_lts_.

These contributions were combined to estimate the expanded uncertainty of the certified value (*u*_CRM_) with a coverage factor *k* as:

4 $$u_{\text{CRM}} = k\; \times \;\sqrt {u_{\text{char}}^{2} + u_{\text{bb}}^{2} + u_{\text{lts}}^{2} }$$

*u*_char_ was estimated as described in Sect. “Characterisation and confirmation”, *u*_bb_ was estimated as described in Sect. “Homogeneity”, *u*_lts_ was estimated as described in Sect. “Stability”.

Due to the sufficient number of degrees of freedom in the different uncertainty calculations, a coverage factor *k* of 2 was applied, to obtain the expanded uncertainties.

Finally, the IRMM-1000a and IRMM-1000b certified value for the production date based on the ^230^Th/^234^U radiochronometer is 09/07/2012 and its expanded uncertainty *u*_CRM_ = 13 day (*k* = 2).

## ILC REIMEP-22

Prior to the release of IRMM-1000a and IRMM-1000b, the EC-JRC-IRMM, in support to the Nuclear Forensics International Technical Working Group (ITWG) and laboratories in the field of nuclear forensics and safeguards, launched a new Regular European Inter-laboratory Measurement Evaluation Programme (REIMEP-22) based on this material, called “U age dating—determination of the production date of a uranium certified test sample”. In total, 11 laboratories participated in REIMEP-22 and reported production dates and uncertainties for the 20 and/or 50 mg certified test samples based on mass- or alpha- spectrometry measurements using the ^230^Th/^234^U radiochronometer. Their results were assessed against the independent reference value in compliance with ISO 13528:2005 and confirmed the abilities of the majority of participating laboratories to determine the ‘age’ of a uranium material containing low amounts of Th [14, 15]. One of the participants in REIMEP-22 reported agreeing production dates based on the ^230^Th/^234^U and the ^231^Pa/^235^U radiochronometers. Although it is a single preliminary result, the agreement within uncertainty of the certified production dates based on both radiochronometers indicates that the complete Pa separation may have also been achieved, so that the production date based on the ^231^Pa/^235^U chronometer could also agree with the ^230^Th/^234^U certified production date. However, further studies are needed to demonstrate the complete Pa separation at the time of preparation of IRMM-1000a and IRMM-1000b and that the material can also serve as a reference for this radiochronometer.

This exercise, the first of this kind within the nuclear forensic community, demonstrated the potential of this new CRM as valuable tool for method validation and calibration.

## Conclusion

The IRMM-1000a and IRMM-1000b uranium reference materials, available in two sizes 20 and 50 mg uranium respectively, were certified according to ISO Guides 34:2009 and 35:2006 for the production date based on the ^230^Th/^234^U radiochronometer. The certified reference value (production date expressed as dd/mm/yyyy) of the IRMM-1000a and IRMM-1000b and its expanded uncertainty was determined as: 09/07/2012 ± 13 day (*k* = 2). The results obtained from the certification study of this CRM and from the ILC REIMEP-22 organised in parallel, demonstrated the successful and complete separation of Th from the original U material to the maximum extent achievable. This confirmed that the “clock” in the reference material was actually set to zero. This novel CRM will contribute to more reliable measurements of the “age” and characterisation of intercepted uranium materials in nuclear forensic and will improve method validation in nuclear safeguards.

## References

[1] ISO Guide 34:2009. (2009). General requirements for the competence of reference material producers

[2] Mayer K, Wallenius M, Varga Z (2013). Nuclear forensic science: correlating measurable material parameters to the history of nuclear material. Chem Rev.

[3] Varga Z, Venchiarutti C, Nicholl A, Krajkó J, Jakopič R, Mayer K, Richter S, Aregbe Y (2015). IRMM-1000a and IRMM-1000b uranium reference materials certified for the production date Part I: methodology, preparation and reference value. J Radioanal Nucl Chem.

[4] ISO/IEC 17025:2005(E). (2005). General requirements for the competence of testing and calibration laboratories

[5] ISO/IEC Guide 98-3:2008. (2008). Evaluation of measurement data—guide to the expression of uncertainty in measurement

[6] ISO Guide 35:2006. (2006). Reference materials—general and statistical principles for certification

[7] De Bievre P, Peiser HS (1997). Basic equations and uncertainties in isotope-dilution mass spectrometry for traceability to SI of values obtained by this primary method. Fresenius J Anal Chem.

[8] Venchiarutti C, Varga Z, Richter S, Nicholl A, Krajko J, Jakopic R, Mayer K, Aregbe Y (2015) Preparation and certification of IRMM-1000a (20 mg) and IRMM-1000b (50 mg). EUR 27146 EN. doi:10.2787/050844

[9] Varga Z, Suranyi G (2007). Production date determination of uranium-oxide materials by inductively coupled plasma mass spectrometry. Anal Chim Acta.

[10] DDEP-BIPM (2004–2013) Table of radionuclides. Monographie BIPM-5. http://www.nucleide.org/DDEP_WG/DDEPdata.htm4

[11] Linsinger TP, Pauwels J, van der Veen A, Schimmel H, Lamberty A (2001). Homogeneity and stability of reference materials. Accrediat Qual Assur.

[12] SoftCRM (2000) Version 2.0.10, SoftCRM2/revised version. Developed by NHRF (G. Bonas, M. Zervou, T. Papaeoannou) and JRC-IRMM (T. Linsinger)

[13] Linsinger TP, Pauwels J, Lamberty A, Schimmel H, van der Veen A, Siekmann L (2001). Estimating the uncertainty of stability for matrix CRMs. Fresenius J Anal Chem.

[14] Venchiarutti C, Varga Z, Richter S, Nicholl A, Krajkó J, Jakopič R, Mayer K, Aregbe Y (2015) REIMEP-22 U age dating—determination of the production date of a uranium certified test sample. EUR27124. doi:10.2787/891879

[15] Venchiarutti C, Varga Z, Richter S, Jakopič R, Mayer K, Aregbe Y (2015). REIMEP-22 inter-laboratory comparison: U age dating—determination of the production date of a uranium certified test sample. Radiochim Acta (accepted)

